# The protective effect of human atrial natriuretic peptide on renal damage during cardiac surgery

**DOI:** 10.1007/s00540-016-2284-0

**Published:** 2016-11-16

**Authors:** Takahiro Moriyama, Shintaro Hagihara, Toko Shiramomo, Misaki Nagaoka, Shohei Iwakawa, Yuichi Kanmura

**Affiliations:** 1 Department of Anesthesiology and Intensive Care, Kagoshima University Hospital, Sakuragaoka 8-35-1, Kagoshima, 46201 Japan; 2 Department of Anesthesiology and Critical Care Medicine, 3-1-1 Maida, Higashi-ku, Fukuoka, Japan

**Keywords:** Acute kidney injury, Cardiac surgery, Human atrial natriuretic peptide, Renin–angiotensin system

## Abstract

**Purpose:**

Acute kidney injury (AKI) is one of the critical complications after cardiac surgery. In the kidney, angiotensin II (Ang II) is formed by independent mechanisms, and activity of the intrarenal renin–angiotensin–aldosterone (RAAS) system contributes to the progression of kidney damage. Although atrial natriuretic peptide (ANP) exerts protective effects against renal injury by inhibiting the RAAS, the mechanisms of this effect have not been completely clarified. We investigated how human ANP (hANP) could prevent renal damage induced by cardiopulmonary bypass.

**Methods:**

Forty-eight patients undergoing cardiac surgery were divided into two groups, with and without hANP infusion. Urinary angiotensinogen, neutrophil gelatinase-associated lipocalin (NGAL) and L-type fatty acid-binding protein (L-FABP) were measured during and after surgery in both groups. Plasma renin activity, Ang II, aldosterone and serum creatinine were also measured.

**Results:**

Urinary angiotensinogen levels in the hANP group were significantly lower than in the non-hANP group after cardiopulmonary bypass surgery, at the end of surgery and 3 h after surgery. At 3 h after surgery, urinary NGAL levels in the hANP and non-hANP groups were 371.1 ± 413.6 and 761.4 ± 437.8 μg/gCr, respectively (*p* < 0.01). Urinary L-FABP levels at the end of surgery in the hANP and non-hANP groups were 238.8 ± 107.4 and 573.9 ± 370.1 μg/gCr, respectively (*p* < 0.01). Moreover, hANP seemed to significantly reduce the incidence of postoperative AKI.

**Conclusions:**

hANP demonstrated renal protective effects during cardiac surgery, and could possibly reduce the incidence of AKI after ischemia–reperfusion surgery. Moreover, this protective effect of hANP is likely induced by inhibition of the intrarenal RAAS.

## Introduction

Acute kidney injury (AKI) is a critical complication that can follow cardiac surgery. Previous studies reported that the incidence of cardiac surgery-associated AKI varies widely from 7 to 40%, depending on the definition [1]. Moreover, the high mortality of such patients has been recognized [2–4]. Cardiopulmonary bypass (CPB)-related injury to the kidneys involves numerous factors, such as hypoxia, vascular endothelial injury, inflammatory reactions, activation of reactive oxygen species (ROS) and the renin–angiotensin–aldosterone system (RAAS). It has also been reported that the incidence of AKI after CPB surgery correlates with a high activity of renin [5]. Atrial natriuretic peptide (ANP), a hormone secreted primarily by atrial cardiomyocytes, is a potent natriuretic, diuretic and vasodilatory peptide that contributes to decreasing blood pressure [6]. Previous studies have found that human ANP (hANP) preserves renal function in patients undergoing coronary artery bypass surgery [7, 8] and abdominal aortic aneurysm repair [9]. One study showed that continuous infusion of hANP during cardiac surgery with CPB maintained postoperative renal function by preventing the activity of the RAAS [7]. Recently, expressions of local RAAS in specific tissues have been recognized, and, in the kidney, intrarenal angiotensin II (Ang II) has been found to be formed by independent mechanisms [10]. In addition, use of urinary angiotensinogen as an index of the activity of the intrarenal RAAS has been assessed [11], and a previous study reported that elevated urinary angiotensinogen was associated with the development of AKI after cardiac surgery [12].

Based on AKI diagnostic criteria, AKI is defined as an increase in serum creatinine (Cr) of ≥0.3 mg/dl or a relative increase of ≥1.5-fold from baseline [13, 14]. Further, even small serum Cr changes were reported to be significantly associated with mortality in a cohort study of cardiac surgery [15]. However, because there is a time lag in the increase of serum Cr following the occurrence of AKI, serum Cr is not an efficient early biomarker for the detection of AKI [16]. Therefore, in the present study, we examined two early urinary biomarkers as indices of AKI: neutrophil gelatinase-associated lipocalin (NGAL) and L-type fatty acid-binding protein (L-FABP). Both NGAL and L-FABP have been recognized as being effective in the detection of kidney injury, although the mechanisms of increasing urinary excretion of these two markers are quite different [17–20].

Previous research reported that the activity of the intrarenal RAAS directly affects the progression of renal disease [10]. In addition, we previously showed in an animal experiment that ANP can prevent the activity of the intrarenal RAAS [21]. Therefore, we hypothesized that ANP exerted renoprotective effects by inhibiting the activity of the intrarenal RAAS, not but the systemic RAAS. The primary endpoint of this study was to investigate whether hANP can prevent the occurrence of AKI during cardiac surgery, and the mechanisms of the renoprotective effects of hANP, with emphasis on the intrarenal RAAS, using NGAL and L-FABP as the biomarkers of AKI.

## Materials and methods

The protocol of this study was approved by the Ethics Committee of Kagoshima University Hospital and registered with the UMIN Clinical Trials Registry (UMIN 000012312) on November 18, 2013. This clinical trial was registered before patient enrollment, and was conducted in accordance with the principles of the Declaration of Helsinki. Prior written informed consent was obtained from each patient.

### Study design and subjects

Forty-eight patients undergoing cardiac surgery with CPB except aortic grafting surgery were enrolled. Exclusion criteria were emergency operation, over 80 years of age, obese patients (BMI > 30 kg/m^2^), low left ventricular function (ejection fraction <40%) and preoperative renal dysfunction (serum Cr level >1.3 mg/dl). After exclusion, all patients were randomly divided into two groups using a computer-based stratified randomization method according to patient’s age, sex and body mass index; one group received an infusion of hANP (carperitide; Daiichi-Sankyo Pharmaceutical Inc., Tokyo, Japan) and the other group was given an infusion of a nutrient-free saline solution in the same manner. We determined the sample size by analyzing the incidence of AKI in our institute based on Kidney Disease: Improving Global Outcomes (KDIGO) criteria.

### Anesthesia and study protocol

Patients were prepared according to standard preoperative procedures after overnight fasting. Morphine hydrochloride (0.2 mg/kg, intramuscularly) was given 30 min before the induction of anesthesia. An intravenous catheter was inserted into a forearm vein, and a cannula was inserted into the radial artery under local anesthesia. Anesthesia then was induced by injecting 0.08 mg/kg midazolam, 5 μg/kg fentanyl and 0.6 mg/kg rocuronium bromide intravenously. Propofol infusion was adjusted to the rate of 4–10 mg/kg/h to maintain bispectral index (BIS; Aspect Medical Systems, Norwood, MA, USA) values within the range of 40–60. The remifentanil infusion was adjusted to the range of 0.1–0.5 μg/kg/min depending on surgical invasiveness. After induction of anesthesia, the lungs were ventilated to normocapnia and red blood cells were transfused to maintain a hematocrit of 25% or more. Infusion of hANP at the rate of 0.025 μg/kg/min or an equivalent volume of a nutrient-free saline solution as placebo was performed via a central venous catheter inserted into the internal jugular vein, from the start of surgery till 12 h postoperatively. CPB was performed with non-pulsatile low-temperature perfusion (target rectal temperature: 34 °C). After surgery, all patients were transferred to the intensive care unit (ICU) and remained sedated for 3 h after surgery. Sedation was then discontinued and the trachea was extubated after confirming hemodynamic stability.

### Measurements

Plasma renin activity, Ang II and aldosterone were measured at five points [before surgery (base line), before and after CPB, at end of surgery, and 3 h after surgery]. At the same time points, urinary angiotensinogen was measured using specific ELISA kits (IBL, Gunma, Japan). Urinary NGAL and L-FABP levels were also determined using specific ELISA kits (Bioporto Diagnostics, Hellerup, Denmark, and CMIC Co. Ltd, Tokyo, Japan, respectively). Urinary angiotensinogen, NGAL and L-FABP were adjusted for urinary creatinine concentrations. Moreover, serum Cr was measured preoperatively and on postoperative days 1, 2 and 3.

### Statistical analysis

Data are expressed as mean ± standard deviation (SD). Patient background and outcome variables between the two groups were compared using *χ* ^2^ tests or unpaired Student’s *t* tests. Other data were analyzed with repeated measures ANOVA using GraphPad Prism software (version 5). We considered *p* < 0.05 to be statistically significant.

## Results

### Patient’s background

Patient characteristics in the two groups are shown in Table 1. There were no significant differences between the two groups. Since hypotension and hypovolemia often occur during cardiac surgery for many reasons, determination of whether hypotension, hypovolemia and electrolyte abnormalities are directly caused by hANP might be difficult. Therefore, we additionally analyzed the data on intraoperative urine output, red blood cell transfusion and fluid balance. We observed that there was no difference in the incidence of complications related to hANP, such as hypotension, hypovolemia and electrolyte abnormalities due to excessive urination, between the hANP and non-hANP groups. If patients developed hypotension, phenylephrine was mainly administered and volume loading was performed. The management of blood pressure was done according to the usual method at our institute, based on the decision of each anesthesiologist. However, no case of serious hypotension occurred because the dose of hANP was low. There was also no rebound phenomenon after discontinuation of hANP administration in the current study.

**Table 1:** Patient demographics

	hANP group (*n* = 24)	Non-hANP group (*n* = 24)
Age (years)	64.7 ± 10.3	65.7 ± 10.9
Sex (male/female)	13/11	15/9
Body weight (kg)	54.2 ± 12.8	57.6 ± 10.9
Preoperative medication		
ARB	13	16
β-Blocker	9	6
Diagnosis		
Coronary artery disease	9	7
Valvular disease	15	17
Operating time (min)	364.7 ± 60.3	374.1 ± 65.1
ACC time (min)	102.2 ± 47.6	110.8 ± 50.1
CPB time (min)	154.7 ± 52.6	161.8 ± 49.1
Intraoperative urine output (ml)	935 ± 125.7	847 ± 91.6
Intraoperative RBC transfusion (ml)	1220 ± 187.3	1176 ± 171.8
Intraoperative fluid balance (ml)	2225 ± 425.3	1805 ± 189.1

There were no significant differences between the two groups

*ARB* angiotensin receptor blocker, *ACC* aortic cross-clamp, *CPB* cardiopulmonary bypass, *RBC* red blood cell

### Activities of systemic and intrarenal renin–angiotensin–aldosterone systems

**Fig. 1:**
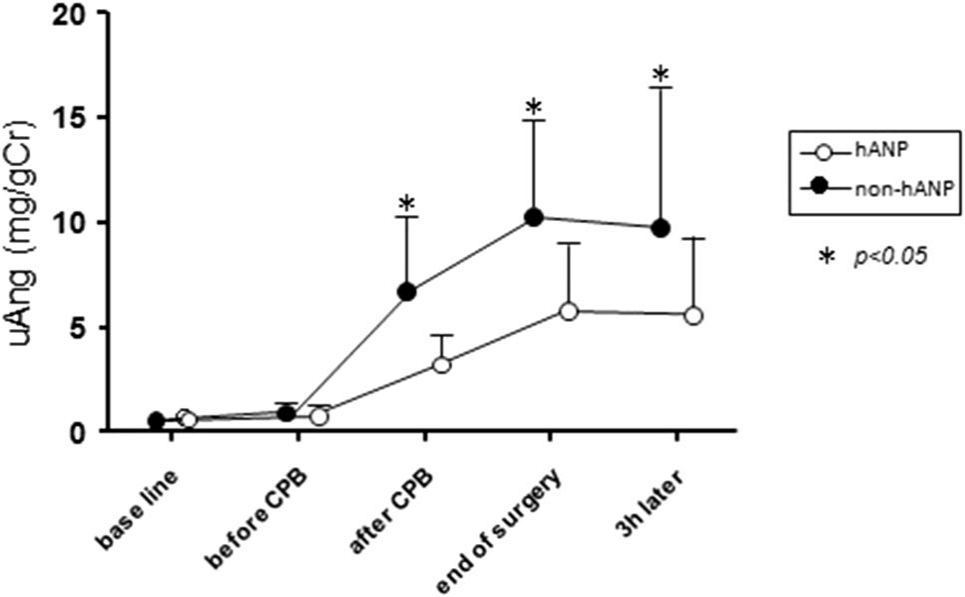
Changes in urinary angiotensinogen from baseline (before surgery) to 3 h after surgery. Urinary angiotensinogen levels were significantly lower in the hANP group compared to the non-hANP group at the time points of after CPB, at end of surgery, and 3 h after surgery. *uAng* urinary angiotensinogen, *CPB* cardiopulmonary bypass

**Fig. 2:**
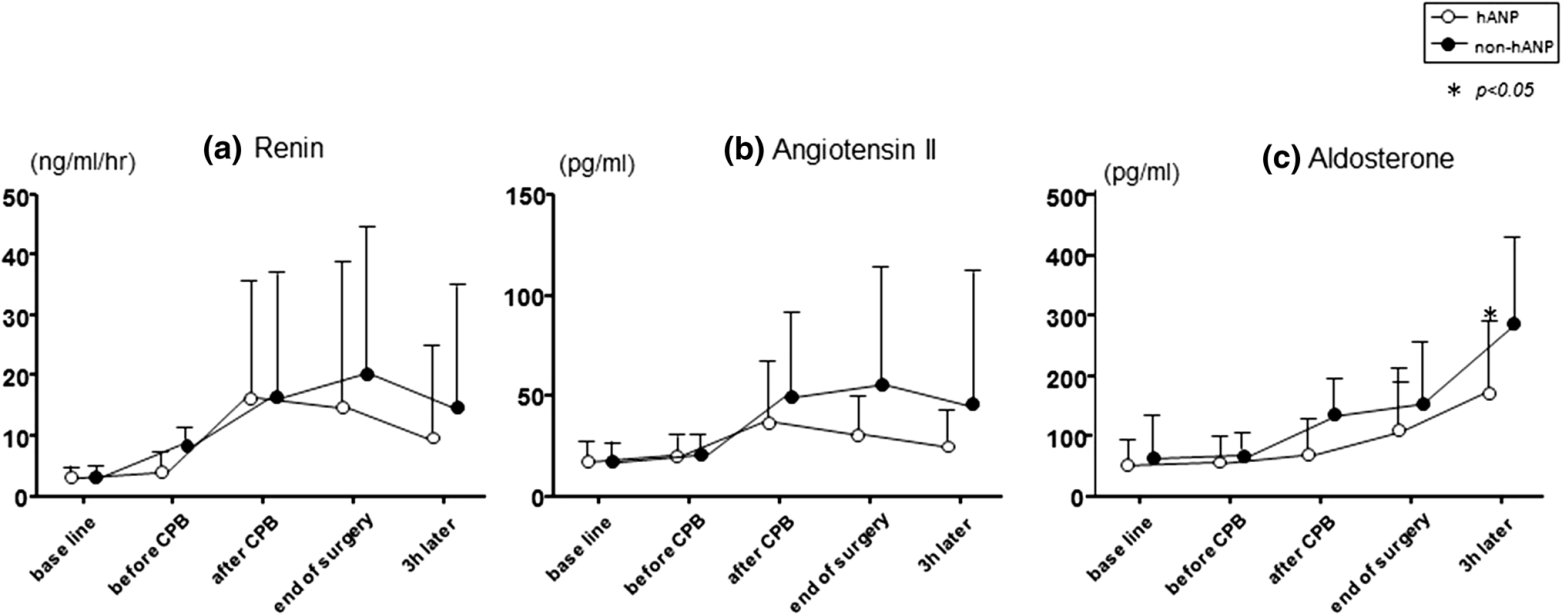
Changes in renin activity (**a**), angiotensin II (**b**) and aldosterone (**c**) from baseline (before surgery) to 3 h after surgery. There were no significant differences in renin activities and angiotensin II levels between the hANP group and the non-hANP group. Aldosterone levels were lower in the hANP group than in the non-hANP group at 3 h after surgery. *CPB* cardiopulmonary bypass

Urinary angiotensinogen increased immediately after CPB compared to baseline in both groups, although urinary angiotensinogen levels were significantly lower in the hANP group than in the non-hANP group after CPB (hANP group 3.3 ± 1.4 mg/gCr versus non-hANP group 6.8 ± 3.6 mg/gCr; *p* = 0.04), at the end of surgery (hANP group 5.8 ± 3.1 mg/gCr versus non-hANP group 10.2 ± 5.1 mg/gCr; *p* = 0.03) and 3 h after surgery (hANP group 5.6 ± 3.9 mg/gCr versus non-hANP group 9.8 ± 6.6 mg/gCr; *p* = 0.04) (Fig. 1). Although renin activity, Ang II and aldosterone levels also started to increase after CPB compared to baseline in both groups, renin and Ang II levels did not differ significantly between the two groups (Fig. 2a, b). Aldosterone in the hANP group was significantly lower than in the non-hANP group only 3 h after surgery (Fig. 2c).

### AKI biomarkers

**Fig. 3:**
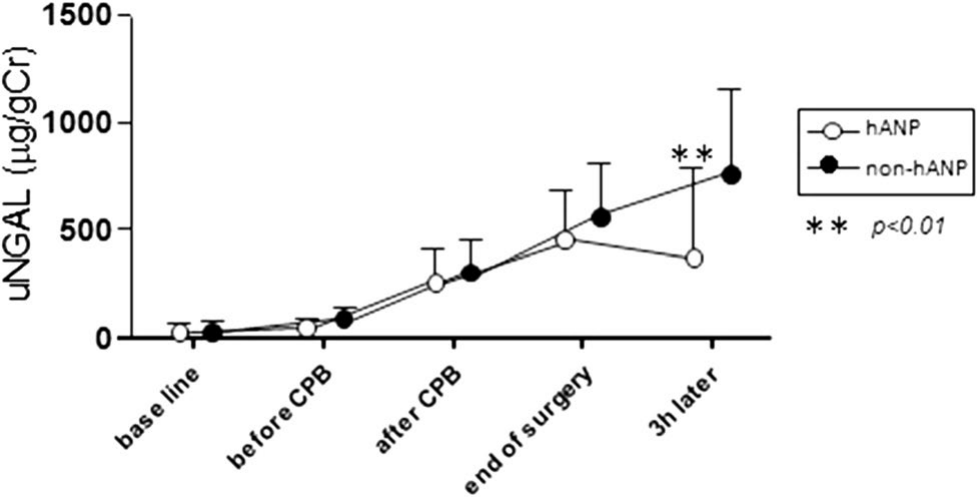
Changes in urinary NGAL from baseline (before surgery) to 3 h after surgery. Urinary NGAL levels were lower in the hANP group than in the non-hANP group at 3 h after surgery. *uNGAL* urinary neutrophil gelatinase-associated lipocalin, *CPB* cardiopulmonary bypass

**Fig. 4:**
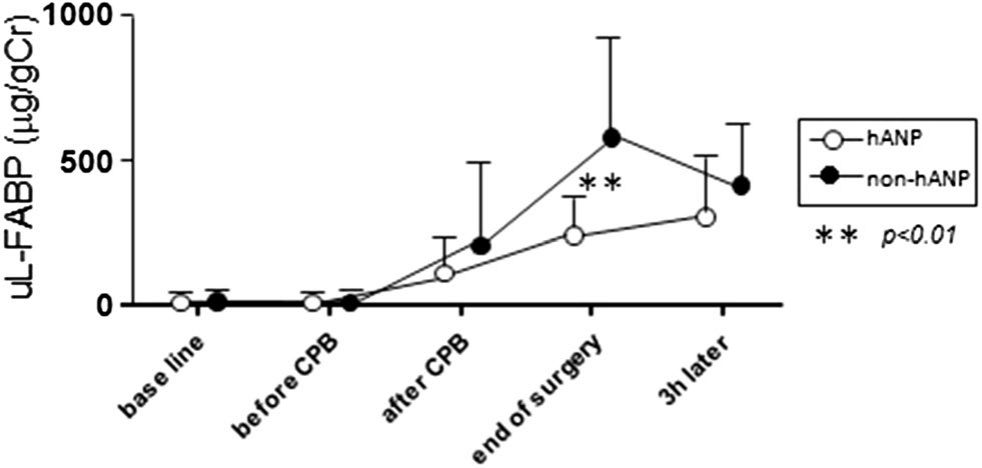
Changes in urinary L-FABP from baseline (before surgery) to 3 h after surgery. Urinary L-FABP levels were lower in the hANP group than in the non-hANP group at the end of surgery. *uL-FABP* urinary L-type fatty acid-binding protein, *CPB* cardiopulmonary bypass

Urinary NGAL and L-FABP levels in the non-hANP group reached a peak 3 h after surgery and at the end of surgery, respectively, with significant differences between the two groups in urinary NGAL and L-FABP levels at these time points. At 3 h after surgery, peak urinary NGAL levels in the hANP group and in the non-hANP group were 371.1 ± 413.6 and 761.4 ± 413.6 μg/gCr, respectively (*p* < 0.01) (Fig. 3). On the other hand, peak urinary L-FABP levels at the end of surgery in the hANP and non-hANP groups were 238.8 ± 107.4 and 573.9 ± 370.1 μg/gCr, respectively (*p* < 0.01) (Fig. 4).

### Serum creatinine and the incidence of postoperative AKI

Serum Cr did not differ significantly between the two groups on postoperative days 1, 2 and 3 (Table 2). However, there were 9 patients in the non-hANP group and 2 patients in the hANP group who developed postoperative AKI, which was diagnosed by an increase in serum Cr of 0.3 mg/dl or higher, based on KDIGO criteria [14]. Hence, the incidence of postoperative AKI was significantly lower in the hANP group (*p* = 0.04).

**Table 2:** Pre- and postoperative serum Cr and incidence of AKI

	hANP group (*n* = 24)	Non-hANP group (*n* = 24)	*p* value
Preoperative sCr (mg/dl)	0.84 ± 0.21	0.86 ± 0.22	0.770
Postoperative day 1 sCr (mg/dl)	0.95 ± 0.42	0.99 ± 0.35	0.719
Postoperative day 2 sCr (mg/dl)	0.98 ± 0.25	1.07 ± 0.34	0.336
Postoperative day 3 sCr (mg/dl)	0.94 ± 0.30	1.02 ± 0.38	0.483
Incidence of AKI (based on KDIGO criteria)	2	9	0.036

*sCr* serum creatinine

## Discussion

The present study demonstrated that low-dose hANP during CPB surgery could prevent an increase in NGAL and L-FABP, which are early biomarkers of AKI, and could reduce the incidence of postoperative AKI. Reportedly, low-dose hANP infusion protects renal function after cardiac surgery via inhibition of systemic RAA activity [7]. However, in the present study, hANP could not completely prevent the increase in serum renin activity and Ang II and aldosterone levels as compared to the control group, all these hormones being activated after CPB in both groups. Recently, the role of the local RAAS in several tissues, such as the kidney, heart, vasculature, adrenal gland, liver, spleen and skeletal muscles, has been emphasized [22]. In the kidney, all components of the renal RAAS are produced independently of the systemic RAAS, and contribute to the progression of both acute and chronic kidney diseases [10]. Intrarenal Ang II can be produced independently from circulating Ang II, its concentrations reportedly being far higher than plasma Ang II levels in AKI, and directly related to the progression of AKI [16]. Moreover, urinary angiotensinogen has been identified as an index of the activity of the renal RAAS [11]. Previous animal experiments demonstrated that hANP attenuated renal injury that was induced by ischemia/reperfusion. The mechanism was attributed to inhibition of Ang II production and intrarenal RAAS activation, which, in turn, prevented the production of urinary angiotensinogen [21]. The present study showed that hANP could significantly prevent the increase in urinary angiotensinogen levels after CPB, as compared to the control group, much earlier than prevention of the increase in both L-FABP and NGAL. On the other hand, hANP could not significantly prevent upregulation of the systemic RAAS, as indicated by plasma renin, Ang II and aldosterone levels, after CPB. Therefore, prevention of the activity of the renal RAAS, but not systemic RAAS, via hANP might inhibit the progress of renal damage after CPB. In a previous study, activation of the systemic RAAS occurred several hours or days later, by which time the biomarkers of AKI had already been upregulated [1]. Increasing activation of the intrarenal RAAS might follow the pattern of systemic renin activity and Ang II and aldosterone concentrations.

The most frequent adverse event associated with hANP infusion is probably hypotension. The optimal dose of hANP for preventing postoperative AKI has not yet been established. We selected low infusion rates of 0.025 μg/kg/min as the dose of hANP in the present study, almost half that used in previous clinical research, which did not result in excessive reductions in blood pressure [7, 8, 23]. Further, since no other noteworthy complications related to hANP infusion were observed, we believe that the dose of 0.025 μg/kg/min of hANP would be safe and efficacious in clinical settings.

Serum Cr has long been used in the diagnosis of kidney disease. However, an increase in serum Cr only indicates renal clearance failure. It has been pointed out that creatinine kinetics are insufficient as an index of acute kidney injury. In the present study, serum Cr did not differ between the two groups on postoperative days 1, 2 and 3, despite the reduced incidence of postoperative AKI with hANP. Hence, we could not demonstrate renoprotective effects of hANP based on an increase in serum Cr, which is contrary to what has been shown in previous randomized controlled studies [7, 8]. Besides, serum Cr does not increase until at least 24–48 h after the development of AKI. Recently, several research groups reported that small increments in serum Cr after cardiac surgery, which was not adopted as a diagnostic criterion for AKI, was significantly associated with the progression of postoperative AKI and mortality [24, 25]. It is possible that hemodilution and hydration may lead to falsely low serum Cr measurements, which in turn can confound the diagnosis of AKI after cardiac surgery. This low accuracy of serum Cr measurements, in addition to the delay in the increase in serum Cr after cardiac surgery, is another drawback of using serum Cr as a marker of AKI. Since serum Cr is not a completely accurate marker in the early diagnosis of AKI, several early and specific biomarkers of AKI are strongly needed.

In the present study, we assessed two typical urinary biomarkers, NGAL and L-FABP, for their utility in the diagnosis of AKI. NGAL is a 25-kDa protein belonging to the lipocalin superfamily, which is secreted by neutrophils [26]. Urinary NGAL has been recognized as an early biomarker of AKI after cardiac surgery [17, 18]. On the other hand, L-FABP is a 14-kDa fatty acid-binding protein, and is a biomarker that is secreted in urine as a result of reactive oxygen stress. The usefulness of L-FABP as a biomarker in the early detection of AKI after cardiac surgery has also been reported [19, 20]. There is a difference between the mechanism of increase in urinary NGAL and L-FABP after CPB. Oxidative stress via renal tissue hypoxia upregulates L-FABP expression and increases the secretion of L-FABP into urine from damaged proximal tubules [27]. In contrast, inflammation induced by various stresses increases serum NGAL, while impaired renal absorption increases urinary NGAL secretion [28]. Therefore, the increase in urinary NGAL levels takes longer than urinary L-FABP levels [27]. In the present study, although both urinary NGAL and L-FABP concentrations increased immediately after CPB in both the non-hANP and hANP groups, hANP prevented the increase in urinary L-FABP at the end of surgery and NGAL 3 h after surgery. Moreover, hANP could significantly prevent the increase in urinary angiotensinogen levels compared to that in the non-hANP group immediately after CPB, well before the increase in both urinary NGAL and L-FABP. Ischemia–reperfusion injury induced by CPB is accompanied by augmentation of the intrarenal RAAS, and the activated intrarenal Ang II promotes renal tissue inflammation, resulting in an increase in urinary NGAL [28]. Ang II also inhibits oxygen delivery and increases oxygen consumption, which could induce the production of ROS [29], which in turn increases the expression and secretion of L-FABP into urine. This suggests that hANP exerts renoprotective effects against inflammation and ROS, which are activated by CPB.

The present study shows that hANP prevents demonstrable activation of the intrarenal RAAS, but not systemic RAAS, which has protective effects against kidney injury. Previous studies measured serum Cr and Cr clearance, which increased 24 h postoperatively and beyond, to evaluate the renoprotective effects of hANP [7, 8, 23]. On the other hand, we demonstrated the possible use of earlier biomarkers, NGAL and L-FABP, in the diagnosis of acute renal damage.

There are several limitations to the present study. One is that we could not directly clarify that hANP prevents an increase in postoperative serum Cr, although it did reduce the incidence of AKI, as determined by serum Cr levels based on the definition of AKI, probably because of the small sample size, although hANP could inhibit the secretion of urinary NGAL and L-FABP. Second, this study was not blinded because carperitide (hANP) has been used as a protective agent during cardiac failure only in Japan, and the utility of hANP in the prevention of AKI or the safety of patients with its administration during surgery has not been completely confirmed. Hence, our institutional ethics committee was not in favor of blinding in the study, although several studies have reported the protective effects of hANP on renal damage [7, 8, 23]. Third, we could not directly prove that hANP prevents activation of the intrarenal RAAS through the secretion of urinary angiotensinogen, which is an indicator of intrarenal RAAS activity. Moreover, hANP inhibited the activity of the systemic RAAS to some extent, although the level of inhibition was not significant. Hence, there is still the possibility that inhibition of the activity of the systemic RAAS by hANP reduced the incidence of postoperative AKI. Further research, including in vivo and in vitro experiments, is needed to clarify the role of hANP in the intrarenal RAAS. In addition, we had intended to decide sample size based on the previous studies. However, the previous studies adopted the values of serum Cr concentrations as the index of AKI after cardiac surgery, while we defined the incidence of AKI based on KDIGO criteria, which adopted the increasing degrees of serum Cr, and there are few reports showing the efficacy of hANP in this manner. Therefore, we could not apply the previous power calculation to the present study for the determination of sample size. Also, we excluded aortic graft surgery from the study based on the advice of our expert statistician, because blood loss, CPB time and operative time were much larger than in other cardiac surgeries. A larger trial including aortic graft surgery with power calculation based on the present results is anticipated.

In conclusion, the efficacy of hANP for prevention of perioperative renal damage was investigated in the present study. hANP exerted renoprotective effects during cardiac surgery, and can possibly reduce the incidence of AKI after ischemia–reperfusion surgery. Moreover, this protective effect of hANP is mainly induced by inhibition of the intrarenal RAAS.

## References

[1] Gaffney AM, Sladen RN (2015). Acute kidney injury in cardiac surgery. Curr Opin Anaesthesiol.

[2] Zanardo G, Michielon P, Paccagnella A, Rosi P, Calo M, Salandin V, Da Ros A, Michieletto F, Simini G (1994). Acute renal failure in the patient undergoing cardiac operation. Prevalence, mortality rate, and main risk factors. J Thorac Cardiovasc Surg.

[3] Corwin HL, Sprague SM, DeLaria GA, Norusis MJ (1989). Acute renal failure associated with cardiac operations. A case-control study. J Thorac Cardiovasc Surg.

[4] Hobson CE, Yavas S, Segal MS, Schold JD, Tribble CG, Layon AJ (2009). Bihorac. Acute kidney injury is associated with increased long-term mortality after cardiothoracic surgery. Circulation.

[5] Myers BD, Moran SM (1986). Hemodynamically mediated acute renal failure. N Eng J Med.

[6] De Bold AJ (1979). Heart atria granularity effects of changes in water-electrolyte balance. Proc Soc Exp Biol Med.

[7] Sezai A, Shiono M, Orime Y, Hata H, Hata M, Negishi N, Sezai Y (2009). Low-dose continuous infusion of human atrial natriuretic peptide during cardiac surgery. Ann Thorac Surg.

[8] Sezai A, Hata M, Niino T, Yoshitake I, Unosawa S, Wakui S, Osaka S, Takayama T, Kasamaki Y, Hirayama A, Minami K (2009). Influence of continuous infusion of low-dose human atrial natriuretic peptide on renal function during cardiac surgery: a randomized controlled study. J Am Coll Cardiol.

[9] Mitaka C, Kudo T, Jibiki M, Sugano N, Inoue Y, Makita K, Imai T (2008). Effects of human atrial natriuretic peptide on renal function in patients undergoing abdominal aortic aneurysm repair. Crit Care Med.

[10] Kobori H, Nangaku M, Navar LG, Nishiyama A (2007). The intrarenal renin-angiotensin system: from physiology to the pathobiology of hypertension and kidney disease. Pharmacol Rev.

[11] Kobori H, Alper AB, Shenava R, Katsurada A, Saito T, Ohashi N, Urushihara M, Miyata K, Satou R, Hamm LL, Navar LG (2009). Urinary angiotensinogen as a novel biomarker of the intrarenal renin-angiotensin system status in hypertensive patients. Hypertension.

[12] Alge JL, Karakala N, Neely BA, Janech MG, Tumlin JA, Chawla LS, Shaw AD, Arthur JMS, AKInet Investigators (2013). Urinary angiotensinogen and risk of severe AKI. Clin L Am Soc Nephrol.

[13] Mehta RL, Kellum JA, Shah SV, Molitoris BA, Ronco C, Warnock DG, Levin A, Acute Kidney Injury Network (2007). Acute Kidney Injury Network: report of an initiative to improve outcomes in acute kidney injury. Crit Care.

[14] Kidney Disease: Improving Global Outcomes (KDIGO) Acute Kidney Injury Work Group. KDIGO clinical practice guideline for acute kidney injury. Kidney Int Suppl. 2012 2:1–138.

[15] Lassnigg A, Schmidlin D, Mouhieddine M, Bachmann LM, Druml W, Bauer P, Hiesmayr M (2004). Minimal changes of serum creatinine predict prognosis in patients after cardiothoracic surgery: a prospective cohort study. J Am Soc Nephrol.

[16] Moran SM, Myers BD (1985). Course of acute renal failure studied by a model of creatinine kinetics. Kidney Int.

[17] Mishra J, Dent C, Tarabishi R, Mitsnefes MM, Ma Q, Kelly C, Ruff SM, Zahedi K, Shao M, Bean M, Mori K, Barasch J, Devarajan P (2005). Neutrophil gelatinase-associated lipocalin (NGAL) as a biomarker for acute renal injury after cardiac surgery. Lancet.

[18] Haase M, Bellomo R, Devarajan P, Schlattmann P, Haase-Fielitz A, NGAL Meta-analysis Investigator Group (2009). Accuracy of neutrophil gelatinase-associated lipocalin (NGAL) in diagnosis and prognosis in acute kidney injury: a systematic review and meta-analysis. Am J Kidney Dis.

[19] Portilla D, Dent C, Sugaya T, Nagothu KK, Kundi I, Moore P, Noiri E, Devarajan P (2008). Liver fatty acid-binding protein as a biomarker of acute kidney injury after cardiac surgery. Kidney Int.

[20] Matsui K, Kamijo-Ikemori A, Sugaya T, Yasuda T, Kimura K (2012). Usefulness of urinary biomarkers in early detection of acute kidney injury after cardiac surgery in adults. Circ J.

[21] Moriyama T, Kanmura Y, Lindahl SG (2016). Atrial natriuretic peptide attenuation of renal ischemia-reperfusion injury after major surgery. J Surg Res.

[22] Abadir PM, Walston JD, Carey RM (2012). Subcellular characteristics of functional intracellular renin-angiotensin systems. Peptides.

[23] Sward K, Valsson F, Odencrants P, Samuelsson O, Ricksten SE (2004). Recombinant human atrial natriuretic peptide in ischemic acute renal failure: a randomized placebo-controlled trial. Crit Care Med.

[24] Lassnig A, Schmid ER, Hiesmayr M, Falk C, Druml W, Bauer P, Schmidlin D (2008). Impact of minimal increases in serum creatinine on outcome in patients after cardiothoracic surgery: do we have to revise current definitions of acute renal failure?. Crit Care.

[25] Liotta M, Olsson D, Sartipy U, Holzmann MJ (2014). Minimal changes in postoperative creatinine values and early and late mortality and cardiovascular events after coronary artery bypass grafting. Am J Cardiol.

[26] Mishra J, Ma Q, Prada A, Mitsnefes M, Zahedi K, Yang J, Barasch J, Devarajan P (2003). Identification of neutrophil gelatinase-associated lipocalin as a novel early urinary biomarker for ischemic renal injury. J Am Soc Nephrol.

[27] Matsui K, Kamijo-Ikemorif A, Sugaya T, Yasuda T, Kimura K (2011). Renal liver-type fatty acid binding protein (L-FABP) attenuates acute kidney injury in aristolochic acid nephrotoxicity. Am J Pathol.

[28] Kuwabara T, Mori K, Mukoyama M, Kasahara M, Yokoi H, Saito Y, Yoshida T, Ogawa Y, Imamaki H, Kusakabe T, Ebihara K, Omata M, Satoh N, Sugawara A, Barasch J, Nakao K (2009). Urinary neutrophil gelatinase-associated lipocalin levels reflect damage to glomeruli, proximal tubules, and distal nephrons. Kidney Int.

[29] Efrati S, Berman S, Abu Hamad R, EI Nakib R, Chanimov M, Siman-Tov Y, Weissgarten J (2012). Hyperglycaemia, inflammation, RAS activation: three culprits to blame for acute kidney injury emerging in healthy rats during general anesthesia. Nephrology.

